# Supplementation With Chinese Medicinal Plant Extracts From *Lonicera hypoglauca* and *Scutellaria baicalensis* Mitigates Colonic Inflammation by Regulating Oxidative Stress and Gut Microbiota in a Colitis Mouse Model

**DOI:** 10.3389/fcimb.2021.798052

**Published:** 2022-01-04

**Authors:** Fan Wan, Mengyu Wang, Ruqing Zhong, Liang Chen, Hui Han, Lei Liu, Yong Zhao, Huiyuan Lv, Fujiang Hou, Bao Yi, Hongfu Zhang

**Affiliations:** ^1^State Key Laboratory of Animal Nutrition, Institute of Animal Science, Chinese Academy of Agricultural Sciences, Beijing, China; ^2^State Key Laboratory of Grassland Agro-Ecosystem, Key Laboratory of Grassland Livestock Industry Innovation, Ministry of Agriculture and Rural Affairs, College of Pastoral Agriculture Science and Technology, Lanzhou University, Lanzhou, China; ^3^Precision Livestock and Nutrition Unit, Gembloux Agro-Bio Tech, University of Liège, Gembloux, Belgium; ^4^Beijing Centre Technology Co., Ltd., Beijing, China

**Keywords:** traditional Chinese medicinal plants, inflammation, gut microbiota, oxidative stress, *Lonicera hypoglauca*, *Scutellaria baicalensis*

## Abstract

Colitis, a chronic inflammatory bowel disease, is characterized by bloody diarrhea and inflammation in the colon. *Lonicera hypoglauca* (“Shanyinhua” in Chinese) and *Scutellaria baicalensis* (“Huangqin” in Chinese) are two traditional Chinese medicinal plants rich in polyphenols, such as chlorogenic acid (CGA) and baicalin (BA), with the effects of anti-inflammation and antioxidation. However, it remains unknown whether extracts from *L. hypoglauca* and *S. baicalensis* (LSEs) could mitigate colonic inflammation. In the present study, ICR mice (22.23 ± 1.65 g) were allocated to three groups treated with chow diet without (CON) or with dextran sulfate sodium (DSS) (CON+DSS) in water or LSE supplementation in diet with DSS (LSE+DSS), and then inflammatory and oxidative parameters and colonic microbiota were detected. The results showed that LSE (500 mg/kg) treatment mitigated DSS-induced colitis symptoms and restored the shortened colon length, the increased disease activity index (DAI), and the damaged intestinal barrier. In serum, LSE supplementation significantly decreased levels of pro-inflammatory cytokines including interleukin (IL)-1β, IL-6, tumor necrosis factor (TNF)-α, and lipopolysaccharide (LPS) and increased IL-10 level. Meanwhile, superoxide dismutase (SOD) and catalase (CAT) were increased, and malondialdehyde (MDA) and reactive oxygen species (ROS) levels were decreased. In the colon tissue, qPCR results showed that LSE supplementation dramatically downregulated the transcriptional expression of *IL-1β*, *IL-6*, *TNF-α*, and *MDA* and upregulated the expression of *SOD1*, *CAT*, and *IL-10*. Additionally, the damaged gut barriers occludin and zonula occludens-1 (ZO-1) in the CON+DSS group were enhanced with LSE supplementation. Furthermore, LSE treatment regulated the gut microbial communities with higher relative abundance of *Dubosiella* and *Ruminococcus torques* group and lower relative abundance of *Bacteroides* and *Turicibacter*. Moreover, the contents of short-chain fatty acids (SCFAs) as products of gut microbiota were also increased. Correlation analysis showed that the mRNA expression of *SOD1* was negatively correlated with *TNF-α* (r = -0.900, *P* < 0.05); the mRNA expression of *IL-6* (r = -0.779, *P* < 0.05) and *TNF-α* (r = -0.703, *P* < 0.05) had a dramatically negative correlation with *Dubosiella*. In conclusion, LSE supplementation could effectively ameliorate inflammation by modulating oxidative stress and gut microbiota in a colitis mouse model.

## Introduction

Inflammatory bowel disease (IBD) has been recognized as a global disease worldwide, especially in Asia, South America, and the Middle East (Manichanh et al., 2012; Kaplan, 2015; Silvio Danese and Panés, 2016; Stournaras et al., 2021). According to the population growth, assuming a 1% prevalence in 2030, there will be over 10 million residents in the western world suffering from IBD over the next decade (Kaplan and Windsor, 2021). Ulcerative colitis (UC), a chronic IBD, is characterized by bloody diarrhea and mucosal inflammation in the colon (Gajendran et al., 2019). The genetic and environmental factors are the major causes of colitis, but the explicit mechanism is still not clear at present (Chow et al., 2009). The main symptom changes of colitis are located in the colon mucosa, recurring inflammatory conditions, and gradually spreading to the entire colon (Zhang et al., 2015). The inflammatory responses and oxidative stress often occur in the pathogenesis and development of UC, which explains that inflammatory infiltration and oxidative damage lead to the occurrence and aggravation of UC (Roessner et al., 2008). Recent research suggested that the possible mechanisms of UC were involved in inflammatory response, oxidative stress, gut barrier dysfunction, gut microbiota dysbiosis, etc. (Martens et al., 2018; Zhai et al., 2019; Yang et al., 2020).

The pathogenesis of colitis was complicated by environmental, genetic, and nutrition-related factors (Lee and Chang, 2021). Among many symptoms of colitis, inflammatory responses and inflammatory infiltration accompanied. During colitis, the mucosal immune system is activated, accompanied by increasing mRNA expressions of pro-inflammatory cytokines (Akanda et al., 2018). The inflammatory responses could be caused by the elevated level of lipopolysaccharide (LPS) in dextran sulfate sodium (DSS)-induced colitis (Mahmoud et al., 2020). Meanwhile, inflammatory infiltration and an uncontrolled immune system could enhance oxidative burden, which are attributed to the continuous overproduction of reactive oxygen species (ROS) (Roessner et al., 2008). In addition, as the first protection of the intestine, the intestinal epithelial barrier consists of the mucous layer, intercellular tight junction (TJ) proteins, and epithelial cells (Martens et al., 2018), which is responsible for the mucosal barrier permeability (Mahmoud et al., 2020). However, colitis often caused gut barrier damage by reducing the expression of TJ proteins zonula occludens-1 (ZO-1), occludin, and claudin-1 (Zhao et al., 2020). Moreover, recent research has indicated that dysfunctional gut microbiota was also involved in the inflammatory responses in colitis (Ni et al., 2017; Friedrich et al., 2019). The relative abundance of *Bacteroides* and *Turicibacter* significantly increased in patients with UC or mice with colitis, and the relative abundance of *Firmicutes* markedly decreased in colitis mice (Gophna et al., 2006; Liu et al., 2020a). In addition, as the microbial metabolites, the levels of short-chain fatty acids (SCFAs) significantly decreased in mice with colitis (Wang et al., 2020b). Numerous studies have also confirmed that enhancing SCFAs could attenuate colitis by reducing pro-inflammatory cytokines (Parada Venegas et al., 2019; Zhao et al., 2020). Therefore, based on the pathogenesis and molecular mechanisms of inflammation in the colon, inhibiting inflammatory response, regulating oxidative stress and gut microbiota structure, and improving gut barrier are considered wise strategies for the alleviation of colitis.

Traditional Chinese medicine, extracted from medicinal plants, including Lonicera hypoglauca and *Scutellaria baicalensis*, showed anti-inflammatory and antioxidative functions (Hu et al., 2020). Studies have shown that medicinal plants may contribute to inflammatory alleviation through inhibiting oxidative stress and regulating the gut barrier and gut microbiota (Almousa et al., 2018; Bai et al., 2019; Xiao et al., 2019). Previous research has indicated that extracts from *Lonicera hypoglauca* could enhance antioxidant capacity in inflammation-related diseases; further action mechanism was thought to be due to the bioactive component chlorogenic acid (CGA) in Lonicera hypoglauca (Liao et al., 2013). CGA was reported to have varieties of pharmacological activities such as antioxidant, anti-inflammatory, and free radical scavenging effects (Ruan et al., 2016; Rui et al., 2017; Lee and Lee, 2018; Li et al., 2018). Further mechanism research in CGA indicated that it can prevent oxidative stress and ameliorate DSS-treated colitis in mice by improving gut microbiota (Gao et al., 2019; Zhang et al., 2019b). Moreover, CGA has been proven to decrease the relative abundance of *Bacteroides* and *Bacteroides*-derived LPS to protect indomethacin-induced colitis and intestinal integrity (Yan et al., 2020). Baicalin (BA) is a polyphenolic compound from *S. baicalensis* extracts, which acts as the main functional ingredient and exerts various pharmacological effects including hepatoprotective, antitumor, antibacterial, anti-inflammatory, antidepressant, and antioxidant activities (Hu et al., 2021). Oral BA (100 mg/kg) could enhance mRNA expression levels of *ZO-1* and *occludin* in 2,4,6-trinitrobenzene sulfonic acid (TNBS)-induced colitis rats (Zhu et al., 2020). In addition, a recent finding demonstrated that BA ameliorated *Mycoplasma gallisepticum*-induced inflammatory damage in the lung by increasing commensal bacterium *Bacteroides fragilis* and modulating phenylalanine metabolism (Yan et al., 2020). Taken together, *L. hypoglauca* and *S. baicalensis* extracts enriched with polyphenol components (especially CGA and BA) may be effective against disorders related to inflammatory responses in colitis.

The beneficial bioactivities of extracts from *L. hypoglauca* and *S. baicalensis* (LSEs) have been studied in various inflammatory models. As to the protective effects of a Chinese medicine product LSE and its related molecular mechanisms in colitis, they are still not clear. Therefore, this study was conducted to evaluate whether LSE could attenuate colitis by inhibiting oxidative stress, improving gut microbiota, and promoting gut barrier.

## Materials and Methods

### Chemicals and Reagents

LSE was purchased from Beijing Centre Biotechnology Co., Ltd. (Beijing, China). DSS (36–50 kDa) was purchased from MP Biomedicals (Irvine, CA, USA). Fluorescein isothiocyanate (FITC)-dextran (70 kDa) was purchased from Sigma-Aldrich (St. Louis, MO, USA). Assay kits, including tumor necrosis factor (TNF)-α, interleukin (IL)-6, IL-1β, IL-10, total antioxidant capacity (T-AOC), glutathione peroxidase (GSH-Px), superoxide dismutase (SOD), catalase (CAT), malondialdehyde (MDA), LPS, and ROS, were purchased from Nanjing Jian Cheng Bioengineering Institute (Nanjing, China). TRIzol reagent was purchased from Invitrogen (Carlsbad, CA, USA). Quantitative real-time polymerase chain reaction (qRT-PCR) was performed using TB Green Premix Ex Taq (TaKaRa, Kusatsu, Japan). The primers used for qPCR were purchased from Sangon Biotech (Shanghai, China). SCFA standards, including acetic, propionic, butyric, isobutyric, isovaleric, and valeric acids, were purchased from Sigma Aldrich (St. Louis, MO, USA).

### High-Performance Liquid Chromatography Analysis

High-performance liquid chromatography (HPLC) analysis of purity of LSE was performed [Waters Alliance E2695, Waters Technology (Shanghai) Co., Ltd., China]. Elution solvents were solvent A (0.4% H_3_PO_4_ in H_2_O) and solvent B (acetonitrile). The gradient step of the solvent was from solvent A to solvent B. The flow rate was 1 ml/min. Detection was performed at 327 nm. The 100-mg LSE detected by HPLC contains 2.29 mg/g CGA and 27.69 mg/g baicalin and are shown in **Figure S1**.

### Animal Care and Experimental Design

The animal experimental protocol described in this study was under the regulations of the Animal Experimental Center of the Institute of Animal Science of the Animal Ethics Committee of the Chinese Academy of Agriculture Sciences according to the Guide for the Care and Use of Laboratory Animals (IAS2020-88).

**Figure 1:**
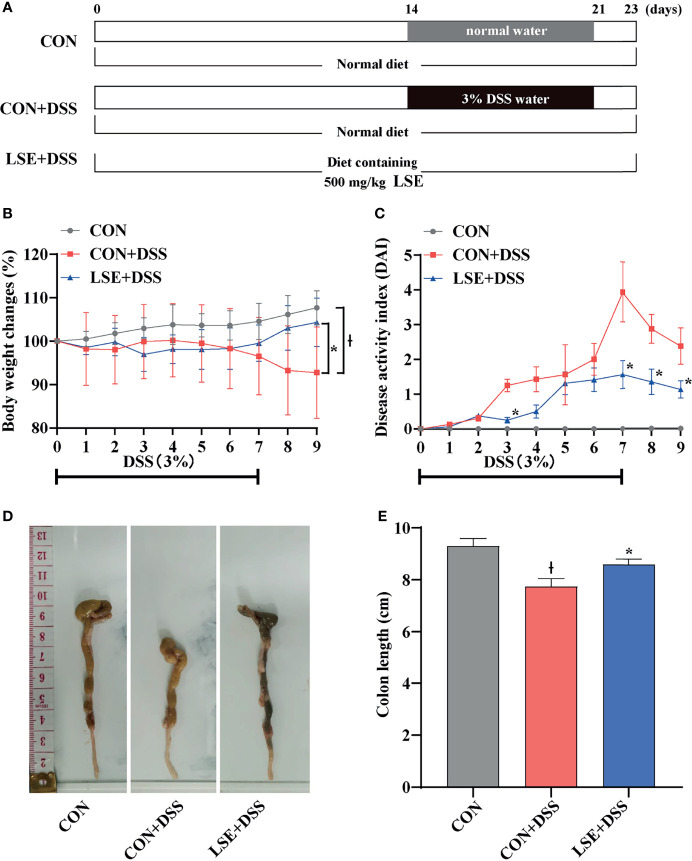
Experimental schedule and basic indicators of LSE on DSS-induced colitis mice. **(A)** Animal treatment schedule. **(B)** Body weight changes. **(C)** DAI score during experimental colitis. *P < 0.05 vs. the DSS-treated group on the same day. **(D)** Images of the colon length. **(E)** Colon length in different treatment groups. * *P <* 0.05 vs. the CON+DSS group, ^Ɨ^ *P <* 0.05 vs. the CON group. Data are presented as mean ± SEM (n = 16 per group). CON (Control group); DSS (Dextran Sulfate Sodium Salt); CON+DSS (CON+DSS group); LSE+DSS (LSE+DSS group). LSE represents extracts from *Lonicera hypoglauca* and *Scutellaria baicalensis*. The same as below.

Female ICR mice (3-week-old) were obtained from Vital River Laboratory Animal Technology (Beijing, China). Under a controlled temperature at 21°C ± 2°C and a 12-h light/dark cycle, all mice had free access to feed and water during the whole experiment. After acclimation for 1 week, mice (body weight 22.23 ± 1.65 g) were randomly divided into three groups (n = 16). Mice fed a basal diet and normal drinking water were the control (CON) group; mice that received a basal diet and drinking water containing DSS were named CON+DSS group; mice fed a basal diet supplemented with 500 mg/kg (w/w) LSE for 23 days and drinking water containing DSS were the LSE+DSS group. Mice in CON+DSS and LSE+DSS groups received normal drinking water for 14 days and then were given 3% DSS in drinking water in days 15–21, followed by 2 days of drinking water without DSS. The experimental design was shown in **Figure 1**. At the end of the experiment, colonic barrier integrity was evaluated by the method of gavage with 70-kDa FITC-dextran in sterile water. Blood samples were collected by orbital blooding, and then the mice were sacrificed by cervical dislocation under anesthesia. The colon length of mice was recorded. The colon tissues were taken out as soon as possible. The proximal colon (2 mm × 6 mm) was stored in 4% paraformaldehyde for histopathological examinations. Colonic contents and remnant colon tissue were frozen in liquid nitrogen for further analysis.

### Evaluation of the Disease Activity Index

To assess colitis statuses, comprehensive disease activity index (DAI) scores of weight loss, stool consistency, and rectal hemorrhage were checked daily (Wang et al., 2020b). Scores were defined as follows: (i) percentage of weight loss: 0 (0%), 1 (1%–5%), 2 (5%–10%), 3 (11%–20%), and 4 (>20%); (ii) stool consistency: 0 (well-formed pellets), 2 (pasty, semi-formed pellets), and 4 (liquid stools); (iii) and rectal bleeding: 0 (no blood), 2 (hemoccult positive), and 4 (gross bleeding).

### Hematoxylin and Eosin Staining and Histopathological Examination

Colon tissues (4 μm) were embedded in paraffin, sectioned, and stained with H&E. Then, histological changes were observed by an optical microscope (Olympus, Tokyo, Japan). Histopathological examination was evaluated based on the infiltration of inflammatory cells and epithelial damage (Yang et al., 2017).

### Serum Inflammatory Cytokines and Antioxidant Capacity Analyses

Serum was obtained by centrifugation at 3,000 rpm for 10 min under 4°C and stored in aliquots at -80℃. The levels of pro-inflammatory cytokines including IL-1β, IL-6, TNF-α, LPS and anti-inflammatory cytokine IL-10 in serum were measured by ELISA (Nanjing Jiancheng Bioengineering Institute, Nanjing, China) according to the manufacturers’ instructions. Meanwhile, the activities of GSH-Px, CAT, and SOD and total antioxidant capacity (T-AOC), as well as the level of MDA and ROS, were measured with corresponding assay kits (Nanjing Jiancheng Bioengineering Institute, Nanjing, China) following the manufacturers’ instructions.

### Colon Permeability

Mice were administered with 100 μl of 100 mg/ml 70-kDa FITC-dextran (Sigma-Aldrich) in sterile water, and blood was collected 4 h before sacrifice (Wang et al., 2020b). Serum was obtained by centrifugation (3,000 rpm, 10 min, 4°C). The concentration of FITC in serum was determined by SynergyH1 automatic microplate reader (Biotek) with excitation wavelength of 485 nm and emission wavelength of 528 nm (Pu et al., 2021).

### Quantitative Real-Time PCR Assay

Total RNA from the colon samples was extracted using TRIzol reagent (Invitrogen, USA), chloroform, isopropanol, and 75% ethanol solution and then treated with DNase I (TaKaRa, China) for possible DNA contamination. The concentration of each RNA sample was quantified using the NanoDrop 2000 (Nanodrop Technologies, USA). The HiFiScript cDNA was generated using the Prime Script RT Master Mix (TaKaRa, China) according to the manufacturer’s instructions. Reverse transcription was conducted at 37°C for 15 min and 85°C for 5 s. qPCR was conducted using the KAPA SYBR FAST qPCR Master Mix kit according to the manufacturer’s instruction. Briefly, 1 μl cDNA template was added to a total volume of 10 μl containing 5 μl KAPA SYBR FAST qPCR Master Mix Universal, 0.4 μl PCR forward primer, 0.4 μl PCR reverse primer, 0.2 μl ROX low, and 3 μl PCR-grade water (KAPA Biosystems, USA). All samples were run in an Applied Biosystems 7500 RT-PCR System (Thermo Fisher Scientific, China). Relative gene expressions were normalized to the housekeeping gene glyceraldehyde-3-phosphate dehydrogenase (GAPDH) and calculated using the 2^-ΔΔCt^ method, where ΔC_t_ = C_t_ (Target) - C_t_ (GAPDH). Primer sequences were designed using Primer 5.0 software and synthesized by Sangon Biotech Co., Ltd. (Shanghai, China). The primers used in this study were listed in **Table 1**.

**Table 1:** Primers used for qPCR assay.

Genes	Forward Primer (5’-3’)	Reverse Primer (5’-3’)
*IL-1β*	TCCTCCTTGCCTCTGATGG	GAGTGCTGCCTAATGTCCC
*IL-6*	GCTGGAGTCACAGAAGGAG	GGCATAACGCACTAGGTTT
*TNF-α*	ACCACCATCAAGGACTCAA	CAGGGAAGAATCTGGAAAG
*IL-10*	GCCATGAATGAATTTGACA	CAAGGAGTTGTTTCCGTTA
*CAT*	TCAGGTGCGGACATTCTA	ATTGCGTTCTTAGGCTTCT
*GPX1*	ATCAGTTCGGACACCAGA	TTCACTTCGCACTTCTCAA
*GPX2*	GTGGCGTCACTCTGAGGAACA	CAGTTCTCCTGATGTCCGAACTG
*SOD1*	GTGAACCAGTTGTGTTGTC	ATCACACGATCTTCAATGGA
*ZO-1*	GAAGAACTGTCAGGCATTG	CATTTACTGGCTGGTATTTT
*Occludin*	ACCGTCTAATCAATCTTTG	AACTCCTGAACCAGCACTC
*NF-κB*	GTGGAGGCATGTTCGGTAG	CTTGGCACAATCTTTAGGG
*p65*	CTTTCGGAGGTGCTTTCGC	CCCTCATGTGCTGGTGTCG
*GAPDH*	GGTCCCAGCTTAGGTTCAT	CAATCTCCACTTTGCCACT

### Gut Microbiota Analysis

Total genome DNA from colonic digesta was extracted using the Fast DNA^®^ SPIN for soil kit (MP Biomedicals, Solon, OH, USA). The quality of the DNA was detected by 1% agarose gel, and DNA was quantified by a NanoDrop 2000 UV-vis spectrophotometer (Thermo Fisher Scientific, Wilmington, DE, USA). The V3-V4 hypervariable region of the bacterial 16S rRNA gene was amplified with PCR using primer pairs 338F (5′-ACT CCTACGGGAGGCAGCAG-3′) and 806R (5′-GGACTACHVGGGTWTCTAAT-3′). The PCR system and amplification conditions were referred to in previous reports (Wang et al., 2020a). PCR-amplified products were extracted from 2% agarose gel and purified using the AxyPrep DNA Gel Extraction Kit (Axygen, Corning, NY, USA) according to the manufacturer’s instruction. After being quantified and purified, amplicons were sequenced. The sequences were analyzed and assigned to operational taxonomic units (OTUs; 97% identity). The products were directly sequenced by an Illumina MiSeq platform (Illumina, SD, USA) (2 × 300, pair end). After being quantified and purified, amplicons were sequenced using Illumina MiSeq platform (Illumina, San Diego, CA, USA) at Majorbio Bio-Pharm Technology Co. Ltd. (Shanghai, China) according to standard protocols. The raw reads were deposited into the NCBI Sequence Read Archive (SRA) database (Accession Number: PRJNA692349).

### Short-Chain Fatty Acid Analysis

The concentrations of SCFAs in colonic contents were measured using Gas Chromatography-Mass Spectrometer (GC-MS). Briefly, colonic content samples were weighed into 1.5-ml centrifuge tubes and mixed with 1 ml ddH_2_O, homogenized, and centrifuged (10,000 rpm, 10 min, 4°C). A mixture of the supernatant fluid and 25% metaphosphoric acid solution (0.9 ml and 0.1 ml, respectively) were vortexed for 1 min and centrifuged (1,000 rpm, 10 min, 4°C) after standing in a 1.5-ml centrifuge tube at 4°C for over 2 h. The supernatant portion was then filtered through a 0.45-μm polysulfone filter and analyzed using Agilent 6890 gas chromatography (Agilent Technologies, Inc., Palo Alto, CA, USA).

### Correlation Analysis

Spearman’s correlation analysis was performed between the colon mRNA expression levels of *CAT*, *SOD1*, *GPX1*, colonic microbiota, and colon mRNA expression levels of *TNF-α*, *IL-6*, *IL-1β*, and *IL-10* based on experimental parameters.

### Statistical Analysis

Data were presented as the mean ± standard error of the mean (SEM). All data were compared by one-way analysis of variance (ANOVA) with Tukey’s test (SPSS 21 software). Spearman’s correlation analysis was performed using RStudio (version 4.0.3) platform. A value of *P* < 0.05 was considered significant, and *P* < 0.01 was considered extremely significant. Plots were performed using GraphPad Prism 8.0.2.

## Results

### LSE Supplementation Mitigated Symptoms of the Colitis

The body weight in the CON+DSS group was significantly decreased compared with that in the CON group, while supplementation with LSE reversed the decreased body weight (*P* < 0.05) (**Figure 1B**). In **Figure 1C**, the DAI score was dramatically increased owing to the DSS treatment, but LSE treatment notably suppressed the increased DAI score. As shown in **Figures 1D, E**, the colon length was shortened in CON+DSS group. However, LSE supplementation significantly inhibited the shortening of the colon induced by DSS (*P* < 0.05). These results implied that supplementation with LSE alleviated DSS-induced colitis symptoms.

### LSE Supplementation Affected Colonic Permeability and Histomorphology

**Figure 2:**
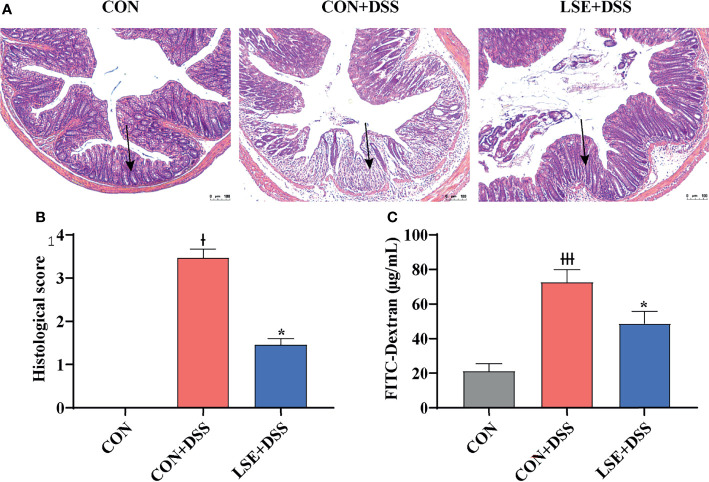
Effects of LSE supplementation on histopathological changes in DSS-induced mouse colon. **(A)** H&E staining images of each group. **(B)** Histopathological score of each group. **(C)** Serum FITC-dextran levels of each group. Data are presented as mean ± SEM, n = 4. * *P* < 0.05 vs. the CON+DSS group, ^†^*P <* 0.05 vs. the CON group, and ^†††^*P* < 0.001 vs. the CON group.

H&E staining results showed that the permeability of the intestinal epithelium was increased in the mucosa and submucosa after DSS treatment (*P* < 0.05). However, LSE supplementation improved the severely damaged histology of the colon (**Figures 2A, B****)**. In addition, colonic barrier integrity was examined by gavage with 70-kDa FITC-dextran in sterile water, and the results showed that the permeability of gut barrier was increased in CON+DSS group, whereas LSE supplementation decreased gut permeability as evidenced by decreased FITC-dextran concentration in serum (*P* < 0.05) (**Figure 2C**).

### LSE Supplementation Altered Levels of Serum Inflammatory Cytokines

**Figure 3:**
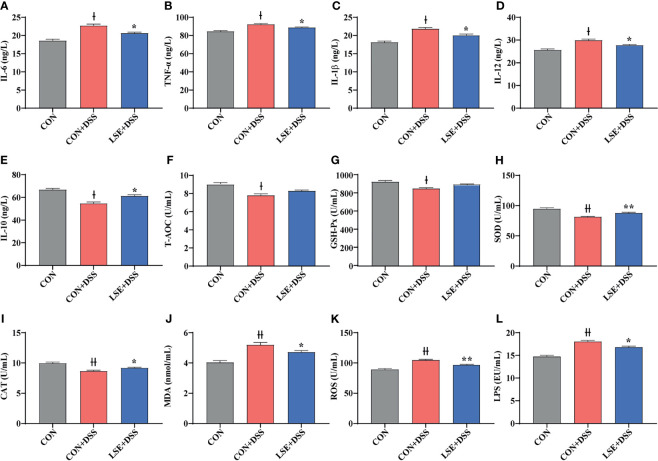
Effects of LSE supplementation on serum inflammation cytokine and antioxidant parameters in DSS-induced colitis mice. **(A)** IL-6. **(B)** TNF-α. **(C)** IL-1β. **(D)** IL-12. **(E)** IL-10. **(F)** T-AOC. **(G)** GSH-Px. **(H)** SOD. **(I)** CAT. **(J)** MDA. **(K)** ROS. **(L)** LPS. Data are presented as mean ± SEM, n = 10. * *P* < 0.05 and **P < 0.01 vs. the CON+DSS group. ^†^*P <* 0.05 and ^††^*P* < 0.01 vs. the CON group.

To investigate the effects of LSE supplementation on cytokine contents in serum of colitis mice, the pro-inflammatory cytokines IL-6, TNF-α, IL-1β, and IL-12 and anti-inflammatory cytokine IL-10 were detected. The results indicated that DSS treatment dramatically increased IL-6, TNF-α, IL-1β, and IL-12 levels compared with the CON group, while LSE treatment reduced levels of pro-inflammatory cytokines IL-6, TNF-α, IL-1β, and IL-12 (**Figures 3A-D**). In addition, mice with DSS-induced colitis exhibited a significant decrease of IL-10 in serum; conversely, LSE treatment significantly relieved inflammatory responses by enhancing IL-10 level (*P* < 0.05) (**Figure 3E**). In this study, we also found an increased level of LPS in CON+DSS group. As expected, LSE supplementation dramatically decreased the production of LPS (**Figure 3L**).

### LSE Altered Gene Expression of Inflammatory Cytokines in the Colon

**Figure 4:**
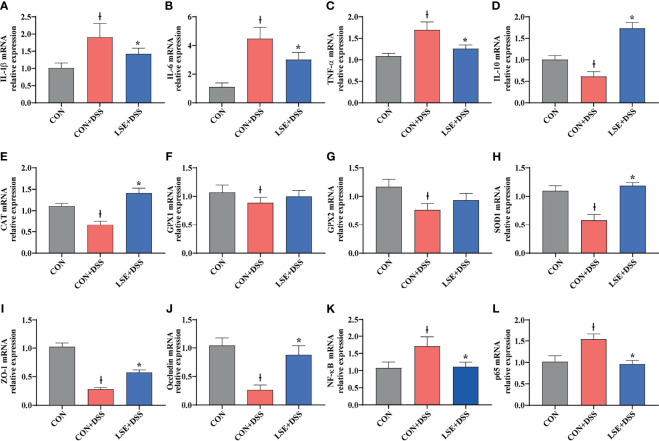
Effects of LSE supplementation on the expression of inflammation cytokine, antioxidant parameters, tight junction proteins, and NF-κB target gene mRNAs in the colon in DSS-induced colitis mice. **(A)** IL-1β. **(B)** IL-6. **(C)** TNF-α. **(D)** IL-10. **(E)** CAT. **(F)** GPX1. **(G)** GPX2. **(H)** SOD1. **(I)** ZO-1. **(J)** Occludin. **(K)** NF-κB. **(L)** p65. Data are presented as mean ± SEM, n = 8. *P < 0.05 vs. the CON+DSS group, ^†^*P <* 0.05 vs. the CON group.

To confirm the anti-inflammatory effect of LSE, the colonic mRNA expressions of inflammatory cytokines *IL-1β*, *IL-6*, *TNF-α*, and *IL-10* were investigated. The results showed that mice in the CON+DSS group had significantly higher relative mRNA levels of *IL-1β*, *IL-6*, and *TNF-α* compared with both the CON and the LSE+DSS groups (*P* < 0.05) (**Figures 4A–C**). Furthermore, LSE+DSS group showed higher mRNA level of anti-inflammatory cytokine *IL-10* than that in CON+DSS group (**Figure 4D**). Additionally, the mRNA expressions of *NF-κB* and *p65* were significantly higher in the CON+DSS group compared to those in the CON group but were markedly lower in the LSE+DSS group than those in the CON+DSS group (*P* < 0.05) (**Figures 4K, L****)**.

### LSE Supplementation Alleviated Oxidative Stress in Serum

In this study, we tested the levels of oxidative stress-related parameters T-AOC, GSH-Px, SOD, CAT, MDA, and ROS. Compared to the CON group, T-AOC and antioxidative enzyme activities including GSH-Px, SOD, and CAT were decreased significantly in the CON+DSS group (*P* < 0.05) (**Figures 3F–I**). However, the enzyme activities of SOD and CAT were increased with LSE administration compared with CON+DSS group (*P* < 0.05) (**Figures 3H, I**). Meanwhile, the level of MDA in serum was significantly higher in the CON+DSS group compared with CON group (*P* < 0.05), but LSE could inhibit this increase induced by DSS (**Figure 3J**). In addition, we found that the level of ROS was increased in CON+DSS group. LSE supplementation significantly decreased the content of ROS (*P* < 0.01) (**Figure 3K**).

### LSE Altered Antioxidant Gene Expression in Colon

To explore the antioxidant effect of LSE, we further tested the colonic mRNA expression levels of *CAT*, *GPX1*, *GPX2*, and *SOD1*. As shown in **Figures 4E–H**, LSE administration significantly enhanced the transcript levels of *CAT* and *SOD1* compared with those in the CON+DSS group (*P* < 0.05). Moreover, LSE enhanced *GPX1* and *GPX2* levels in DSS-induced colitis mice, although it was not significant (*P* > 0.05).

### LSE Supplementation Enhanced the Gene Expressions of Tight Junction Proteins

To further confirm the protective effects of LSE supplementation in the colon, TJ proteins were tested. The mRNA expressions of *ZO-1* and *occludin* were significantly lower in the CON+DSS group compared with those in the CON group (*P* < 0.05). The mRNA expressions of *ZO-1* and *occludin* were markedly higher in the LSE+DSS group than those in the CON+DSS group (**Figures 4I, J**).

### LSE Supplementation Modulated the Composition of Colonic Microbiota

**Figure 5:**
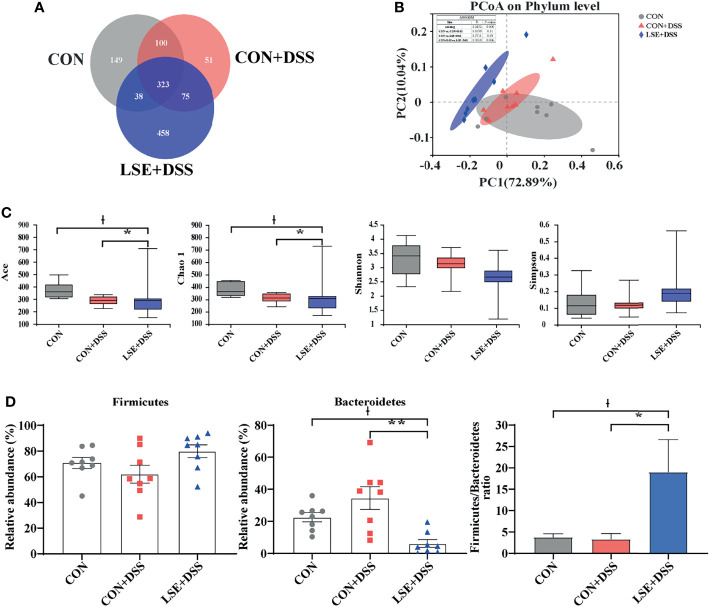
Effects of LSE administration on intestinal microbiota inflammatory in DSS-induced colitis mice. **(A)** A Venn diagram showing the overlap of the OTUs identified in the intestinal microbiota among the three groups. **(B)** PCoA plot of the gut microbiota based on weighted UniFrac distance. **(C)** The α-diversity of each group. **(D)** The abundance of *Firmicutes* and *Bacteroidetes* and *Firmicutes*/*Bacteroidetes* ratio at phylum levels. Data are presented as mean ± SEM, n = 8. * *P* < 0.05 and ** *P* < 0.01 vs. the CON+DSS group, ^†^*P <* 0.05 vs. the CON group.

Using 16S rRNA amplicon sequencing, the microbiota in the colonic content was analyzed. Each sequence length was 401–440 base pairs. The Venn diagram showed that mice in the CON, CON+DSS, and LSE+DSS groups contained 323 common OTUs and 149, 51, and 458 unique OTUs, respectively (**Figure 5A**). The β-diversity was conducted by principal coordinate analysis (PCoA) based on weighted UniFrac metrics, and the results showed that the gut microbiota in the LSE+DSS group was significantly different from that in the CON and CON+DSS groups (*P* < 0.05) (**Figure 5B**). The α-diversity results showed that there was a significant difference in the indexes of ACE and Chao1 among the three groups (*P* < 0.05) (**Figure 5C**). At the phylum level, the predominant bacterial communities were *Firmicutes* and *Bacteroidetes* in mice (**Figure 5D**). In contrast to CON+DSS group, there was a significant decrease in the relative abundance of *Bacteroidetes* in LSE+DSS group (*P* < 0.05). Moreover, the *Firmicutes*/*Bacteroidetes* ratio was significantly higher in the LSE+DSS group compared with those in the CON and CON+DSS groups (*P* < 0.05) (**Figure 5D**).

**Figure 6:**
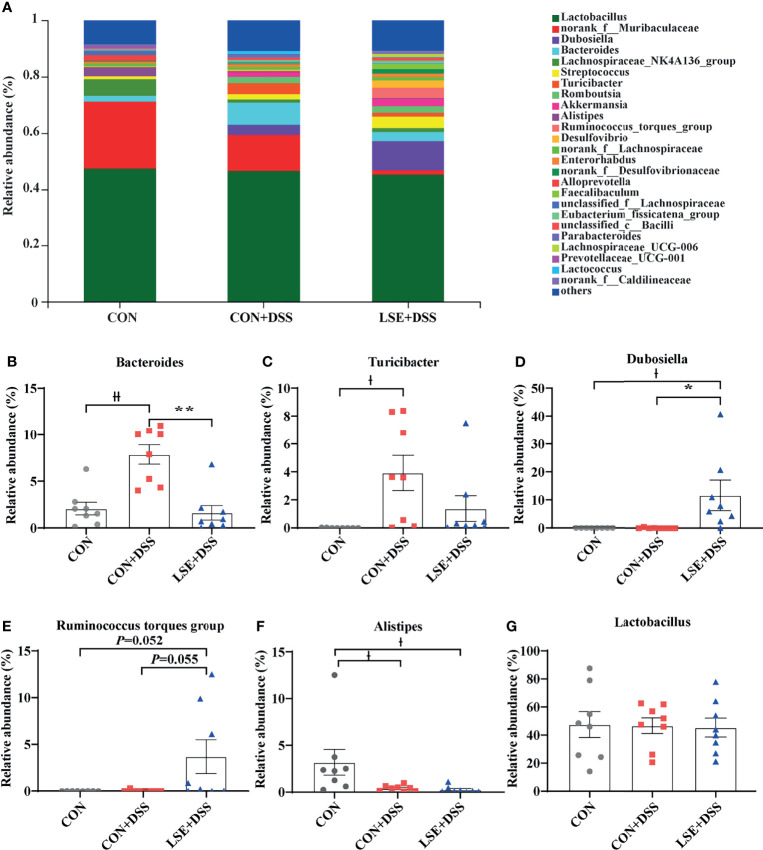
Effects of LSE administration on intestinal microbiota inflammatory in DSS-induced colitis mice. **(A)** Structural comparison of intestinal microbiota between LSE+DSS, CON+DSS, and CON groups at genus levels. **(B)** Relative abundance of *Bacteroides*. **(C)** Relative abundance of *Turicibacter*. **(D)** Relative abundance of *Dubosiella*. **(E)** Relative abundance of *Ruminococcus torques* group. **(F)** Relative abundance of *Alistipes*. **(G)** Relative abundance of *Lactobacillus*. Data are presented as mean ± SEM, n = 8. * *P* < 0.05 and ** *P* < 0.01 vs. the CON+DSS group, †*P <* 0.05 and ^††^*P* < 0.01 vs. the CON group.

At the genus levels, *Bacteroides*, *Turicibacter*, *Dubosiella*, *Ruminococcus torques* group, and *Alistipes* were bacteria with different contents. *Bacteroides* (7.87%) and *Turicibacter* (3.93%) were important bacterial genera in the CON+DSS group, while there was a significantly decreased relative abundance of *Bacteroides* (*P <* 0.01) in the CON and LSE+DSS groups separately (**Figures 6A, B**). In addition, the relative abundance of *Turicibacter* in the CON group was decreased compared with that in the CON+DSS group significantly (*P <* 0.05) but not significantly with that in the LSE+DSS group (*P >* 0.05) (**Figure 6C**). *Dubosiella* (11.71%) and *Ruminococcus torques* group (3.69%) were important bacterial genera in the LSE+DSS group, but the CON+DSS group showed lower relative abundance of *Dubosiella* (*P <* 0.05) and *Ruminococcus torques* group (*P* = 0.055) (**Figures 6D, E**). Similar results were found in the CON group. The CON group showed a higher relative abundance of *Alistipes* than that in the CON+DSS (*P <* 0.05) and LSE+DSS groups (*P <* 0.05) (**Figure 6F**). The relative abundance of *Lactobacillus* was not significant in all groups (**Figure 6G**).

To identify the key microbial communities among the three groups, the linear discriminant analysis effect size (LEfSe) and linear discriminant analysis (LDA) were performed. In this study, there were distinct species in the CON, CON+DSS, and LSE+DSS groups (**Figures S2A, B**). The results demonstrated that *Bacteroides*, *Turicibacter*, *Actinobacteria*, *Acholeplasmatales*, *Eubacterium*, *Staphylococcales*, *Enterobacterales*, and *Rhodespirillales* were dominant bacteria in the CON+DSS group, while *Dubosiella*, *Erysipelotrichales*, *Desulfovibrionia*, *Peptostreptococcaceae*, and *Patescibacteria* were identified as the dominant bacteria in the LSE+DSS group.

### LSE Supplementation Influenced Short-Chain Fatty Acids

**Figure 7:**
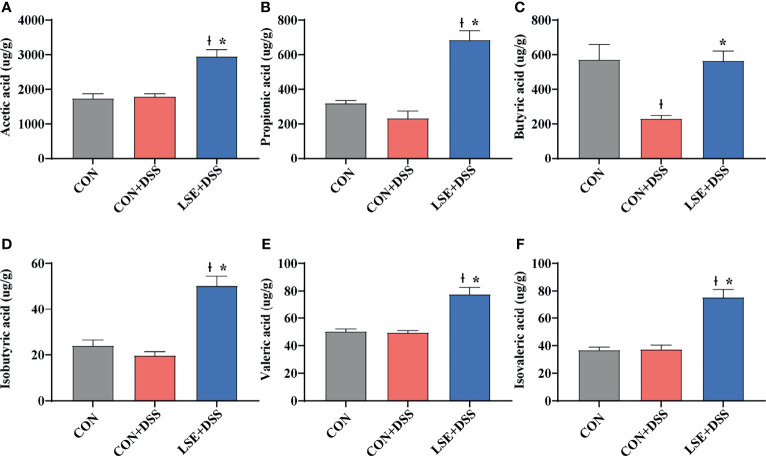
Effect of LSE administration on SCFA production in DSS-induced colitis. **(A)** Acetic acid. **(B)** Propionic acid. **(C)** Butyric acid. **(D)** Isobutyric acid. **(E)** Valeric acid. **(F)** Isovaleric acid. The values are expressed as mean ± SEM, n = 8. * *P* < 0.05 vs. the CON+DSS group, †*P <* 0.05 vs. the CON group.

As metabolites of gut microbiota, SCFAs can protect the intestine by alleviating inflammation and maintaining the integrity of the intestinal epithelial cells. The results of SCFAs in the colonic content showed that there was a significant decrease in butyric acid in the CON+DSS group compared with that in the CON group. Importantly, the levels of acetic, propionic, butyric, isobutyric, valeric, and isovaleric acid in the LSE+DSS group were significantly improved compared with the CON+DSS group (**Figure 7**).

### Correlation Analysis Between Oxidative Indexes, Microorganisms, and Inflammatory Cell Cytokines in Dextran Sulfate Sodium-Induced Colitis Mice

**Figure 8:**
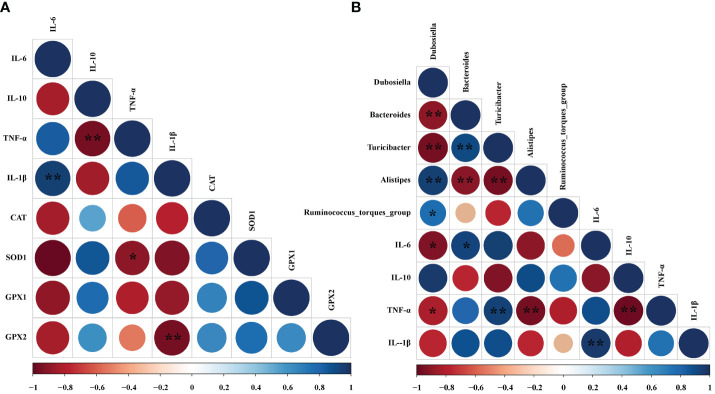
Correlation analysis between oxidative indexes, microorganisms, and inflammatory cell cytokines in DSS-induced colitis mice. **(A)** Correlation analyses between the oxidative indexes and colon mRNA expression of inflammatory cytokines. **(B)** Correlation analyses between the colonic microbiota and colon mRNA expression of inflammatory cytokines. The color of the circles demonstrated the level of the correlation index, and the size of the circles represents the strength of correlation (large size = stronger correlation). The * *P* < 0.05 and ** *P* < 0.01 represents correlation with each other.

To find whether the inflammatory parameters were correlated with the colonic microbiota, colonic SCFAs, and oxidation-related index, the Spearman’s correlation analysis was carried out based on experimental parameters. The results indicated that there was a significantly negative correlation between SOD1 and TNF-α (r = -0.900, *P* < 0.05). And GPX2 was negatively correlated with IL-1β (r = -0.814, *P* < 0.05) (**Figure 8A**). In addition, the mRNA expressions of *IL-6* (r = -0.779, *P* < 0.05) and *TNF-α* (r = -0.703, *P* < 0.05) had dramatically negative correlations with *Dubosiella*. However, *IL-6* mRNA expression exhibited a significantly positive correlation with *Bacteroides* (r = 0.738, *P* < 0.05). Moreover, the mRNA expression of *TNF-α* had a significantly positive relationship with *Turicibacter* (r = 0.671, *P* < 0.01). Conversely, the mRNA expression of *TNF-α* was notably negatively correlated with *Alistipes* (r = -0.770, *P* < 0.01) (**Figure 8B**).

## Discussion

UC is a complex and recurrent colonic inflammatory disease with an increased incidence worldwide (Stournaras et al., 2021). DSS-induced colitis is a classic UC model in mice because of the similar symptoms in patients with UC (Kaplan, 2015). The major indicators of DAI, the colon length, and the ratio of colon length/body weight have shown that DSS exposure can induce classic symptoms of colitis (Ma et al., 2018; Zhao et al., 2019; Dong et al., 2020; Zhao et al., 2020). Over the years, components from Chinese medicinal plants *L. hypoglauca* and *S. baicalensis* were widely used for inflammatory diseases, including upper respiratory tract infection, acute and chronic tonsillitis, acute and chronic pharyngitis, and burn infection (Marafini et al., 2019; Hu et al., 2020). In the present study, the colitis mouse model was established by drinking 3% DSS water for 7 days. We have indicated that LSE supplementation could alleviate DSS-induced colitis by inhibiting the inflammatory response, which was potentially associated with improved gut microbiota community and redox state.

Inflammatory response plays a key role in the activation of IL-6, IL-1β, and TNF-α in colitis (Marafini et al., 2019). IL-10 is a necessary inflammatory factor for inducement and maintenance of regulatory T (Treg) cells against colitis (Sivaprakasam et al., 2016). Numerous plant-extracted polyphenols can decrease the levels of inflammatory cytokines to attenuate inflammation responses (Sandoval-Ramirez et al., 2021). For example, caffeic acid, tea polyphenols, salvianolic acid, and sesamol could ameliorate colitis by reducing the levels of IL-1β, IL-6, TNF-α, and other pro-inflammatory cytokines in DSS-induced models (Ye et al., 2009; Wang et al., 2018; Liu et al., 2020b; Zhao et al., 2020). Major bioactive phenolics (CGA) of *Berberis lycium* Royle fruit extract (BLFE) could enhance IL-10 level in the attenuation of LPS-induced inflammatory responses (Sharma et al., 2020). Flavonoid-enriched extract from *S. baicalensis* root (FESR) modulated the inflammatory responses by increasing the level of IL-10 in influenza A virus-induced acute lung injury in mice, which was speculated to be due to BA, the most abundant compound in FESR (Zhi et al., 2019). In addition, BA, the major ingredient of *S. baicalensis*, could inhibit the production of IL-6 and TNF-α but increase the level of IL-10 in serum after methicillin-resistant *Staphylococcus aureus* (MRSA)-stimulated inflammatory response in mice (Shi et al., 2020). Similarly, in the present study, we also found that the levels of serum IL-1β, IL-6, and TNF-α were increased in DSS-induced colitis, while LSE supplementation could decrease these inflammatory cytokines. Besides, LSE administration could reverse the decreased level of IL-10 caused by DSS. Therefore, these results of inflammatory parameters showed that LSE supplementation could enhance anti-inflammatory capacity to relieve colitis in DSS-induced mice.

The gut barrier function consists of TJ proteins and adherens junction, which form a physical barrier to inhibit inflammatory infiltration and protect gut health (Turner, 2009). The barrier is broken when the intestinal TJ proteins (ZO-1, occludin, and claudin-1) of epithelium cells are disrupted (Grosheva et al., 2020). Recent research has reported that the deteriorative colonic epithelial disruption and inflammatory infiltration usually occurred in UC patients and colitis mice (Marafini et al., 2019; Grosheva et al., 2020; Zhao et al., 2020). In this study, we found a decreased histological score in DSS group, while LSE supplementation reversed the decrease. Especially, in the LSE+DSS treatment, the FITC-dextran concentration of serum was notably lower than that of DSS treatment. Furthermore, the loss of intestinal epithelial ZO-1 and occludin triggered inflammatory infiltration in DSS group, while LSE supplementation in the diet reversed these alterations. The results were similar with previous studies. Therefore, we speculated that LSE supplementation played a protective effect on the gut integrity and effectively attenuates elevation in gut permeability.

Oxidative stress is involved in a large number of chronic inflammatory diseases such as UC (Piechota-Polanczyk and Fichna, 2014). A recent study manifested that Divya-Swasari-Kwath, herbal decoction prescribed in India, could inhibit asthma in mice by increasing the levels of CAT, GSH, and SOD probably due to CGA that existed in herbal decoction Divya-Swasari-Kwath (Balkrishna et al., 2020). Moreover, CGA had been indicated to prevent cadmium-induced oxidative damage in the liver and kidney tissues by reducing the activation of myeloperoxidase (MPO) (Ding et al., 2021). Besides, BA also could inhibit oxidative stress. For example, BA could against oxygen-glucose deprivation/reoxygenation (OGD/R) in a neuron–astrocyte coculture system by suppressing oxidative stress, inflammation, and apoptosis (Li et al., 2021). BA also inhibits oxidative stress to alleviate the development of atherosclerosis by decreasing the level of MDA and enhancing the levels of SOD, CAT, and GSH-Px (Xin et al., 2020). In this study, we also showed that LSE supplementation could effectively ameliorate DSS-induced colitis mice by inhibiting oxidative stress. We hypothesized that the result was partly attributable to the functional components CGA and BA in LSE. Above all, the protective effect of LSE on colitis has also been enhanced through alleviating oxidative stress in our study.

In the process of the development of colitis, the intestinal microbiota structure plays a vital role. A recent study showed that gut microbiota dysbiosis could destroy the gut epithelium barrier and cause the infiltration of inflammatory cytokines (Caruso et al., 2020). However, CGA supplementation could modulate colonic bacterial growth and reproduction to attenuate DSS-induced colitis in mice in a previous study (Zhang et al., 2019b). In addition, oral BA could mitigate TNBS-induced rat colitis by improving gut microbiota (Zhu et al., 2020). Therefore, we guessed that LSE supplementation may alter gut microbiota to alleviate DSS-induced colitis. The effect of colonic microbial was analyzed by sequencing of 16S rRNA after LSE supplementation. The data showed that LSE supplementation decreased the Ace and Chao1 indexes, suggesting that LSE lowered the α-diversity of colonic microbiota, which might be due to the antibacterial effect of LSE. A previous study showed that the relative abundance of *Bacteroides* was increased in patients with UC or mice with colitis, and the relative abundance of *Firmicutes* markedly decreased in colitis mice (Gophna et al., 2006; Liu et al., 2020a). Consistent with this, at the phylum level, we found that the relative abundance of *Bacteroidetes* was significantly increased by DSS treatment, which can be suppressed with LSE treatment. Furthermore, the ratio of *Firmicutes*/*Bacteroidetes* was significantly increased by LSE supplementation compared with CON and CON+DSS groups, which may be one of the reasons for the improved colitis symptom. *Dubosiella* was regarded as potentially beneficial bacteria to fight against UC (Zhai et al., 2019). At the genus level, we found that the proportion of *Dubosiella* was significantly increased by LSE supplementation compared with DSS group. But the mechanism is not distinct. Therefore, more research is needed to explore the protective mechanisms of *Dubosiella* for intestinal inflammation. The relative abundance of *Ruminococcus torques* group was increased after metformin treatment, which improved overweight/obese adults (Mueller et al., 2021). It means that *Ruminococcus torques* group could alleviate obesity. In our study, we found that the improved colitis was linked to a higher *Ruminococcus torques* group, demonstrating that *Ruminococcus torques* group might have another application as a potential probiotic in anti-inflammatory responses. In addition, previous different studies had shown that the fraction of the harmful bacteria *Turicibacter* was higher in DSS-treated mice (Wan et al., 2019; Liu et al., 2020a). In our study, LSE supplementation could decline the abundance of *Turicibacter*. Therefore, we confirmed that LSE supplementation had a remission effect on DSS-induced colitis by improving gut microbiota. In addition, we also found that the relative abundance of some specific bacteria was increased after LSE supplementation in DSS-induced colitis mice, which provided us a promising approach for the future development of probiotics in the gut.

SCFAs, as gut microbiota-derived metabolites, are able to promote the activation of T-cell functions in the intestinal mucosal tissue through the activation of G protein-coupled receptors and *via* epigenetic effects through inhibition of histone deacetylase (Park et al., 2015; Ni et al., 2017). In the present study, LSE supplementation enhanced SCFA levels, including acetic acid, propionic acid, butyric acid, isobutyric acid, valeric acid, and isovaleric acid. This is a new discovery. In the previous study, *Ruminococcus torques* group was positively correlated with SCFA levels by researching some people who ingested less starch in order to reduce body weight (Zhang et al., 2019a). In the present study, the enhanced SCFA levels may be associated with increased abundance of *Ruminococcus torques* group after LSE supplementation. This process improved reconversion of immune tolerance and inhibited inflammatory responses in colitis mice (Smith et al., 2013). The specific mechanism of butyric acid and acetic acid was to inhibit G protein-coupled receptors 43 to inhibit histone deacetylase in Treg cells, playing an anti-inflammatory role (Berer and Krishnamoorthy, 2014; Sivaprakasam et al., 2016). Butyric acid and acetic acid were reduced in feces of UC patients (Machiels et al., 2014). However, various studies have demonstrated that supplementation with Chinese medicine extracts, such as *Hericium erinaceus* extract and salvianolic acid B, could increase the production of acetic acid and butyric acid subsequently to alleviate inflammation including colitis (Wu et al., 2018; Shao et al., 2019). In the present study, LSE supplementation can markedly enhance SCFA levels. These data indicated that the beneficial effect of LSE supplementation on colitis was mechanistically possibly attributable to the effect of SCFAs. However, further investigation needs to be explored.

**Figure 9:**
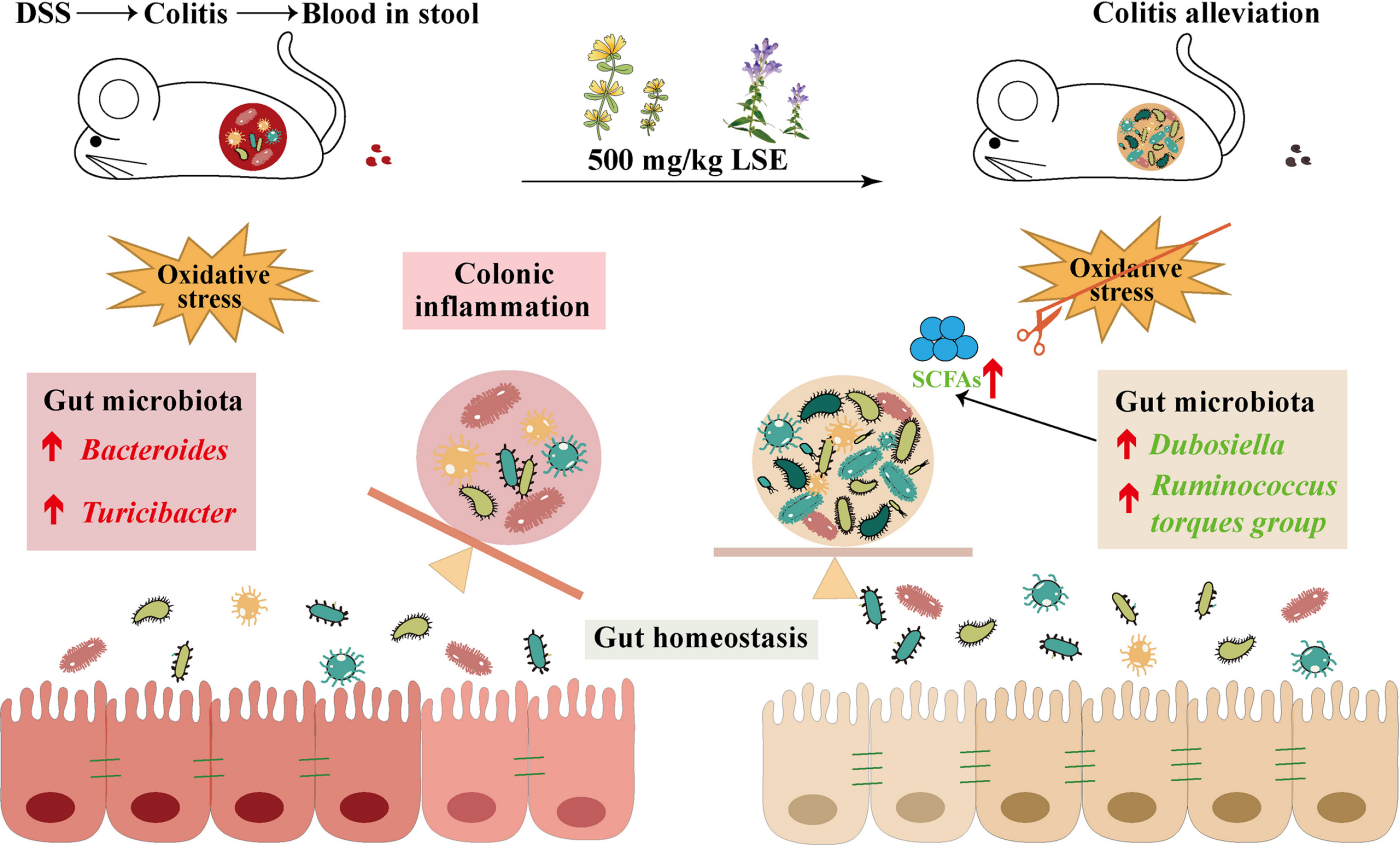
LSE supplementation can alleviate colonic inflammation by regulating oxidative stress and gut microbiota community in DSS-induced colitis mice: a possible mechanism.

In conclusion, our data provide the evidence that LSE supplementation in diet can inhibit inflammatory responses and oxidative stress, modulate the dysbiosis of gut microbiota, and enhance metabolite SCFA levels in DSS-induced colitis mice (**Figure 9**). Furthermore, it is reasonable to conclude that CGA and BA in LSE play major biological functions. More research is needed to explore the precise mechanisms and components of LSE in the future.

## Data Availability Statement

The datasets presented in this study can be found in online repositories. The names of the repository/repositories and accession number(s) can be found here: https://www.ncbi.nlm.nih.gov/, PRJNA750729.

## Ethics Statement

The animal study was reviewed and approved by the Animal Ethics Committee of the Institute of Animal Science, Chinese Academy of Agriculture Sciences.

## Author Contributions

FW, BY, LC, RZ, YZ, HL, FH, and HZ conceived and designed the experiments. FW and HH performed the experiments. FW, MW, and BY analyzed the data. LL contributed to the reagents/materials/analysis tools. FW wrote the paper. All authors read and approved the final article.

## Funding

This work was supported by the Central Public-Interest Scientific Institution Basal Research Fund (Y2021GH01-4), State Key Laboratory of Animal Nutrition (2004DA125184G2102), the Major Scientific Research Tasks for Scientific and Technological Innovation Projects of the Chinese Academy of Agricultural Sciences (CAAS-ZDRW202006-02), and the China Agriculture Research System (CARS-41). The funder had the following involvement in the study: “supplementation with Chinese medicinal plant extracts from Lonicera hypoglauca and Scutellaria baicalensis mitigates colonic inflammation by regulating oxidative stress and gut microbiota in a colitis mouse model”.

## Conflict of Interest

HL is employed by Beijing Centre Technology Co., Ltd., Beijing.

The remaining authors declare that the research was conducted in the absence of any commercial or financial relationships that could be construed as a potential conflict of interest.

## Publisher’s Note

All claims expressed in this article are solely those of the authors and do not necessarily represent those of their affiliated organizations, or those of the publisher, the editors and the reviewers. Any product that may be evaluated in this article, or claim that may be made by its manufacturer, is not guaranteed or endorsed by the publisher.

## Abbreviations

CAT, catalase; CD, Crohn’s disease; DAI, disease activity index; DSS, dextran sulfate sodium; GSH-Px, glutathione peroxidase; Gpx1, glutathione peroxidase 1; Gpx2, glutathione peroxidase 2; H&E, hematoxylin and eosin; HO-1, heme oxygenase-1; IBD, inflammatory bowel disease; IL-1β, interleukin-1 beta; IL-6, interleukin-6; IL-10, interleukin-10; IL-12, interleukin-12; LPS, lipopolysaccharide; MDA, malondialdehyde; NF-κB, nuclear factor-kappaB; NQO1, NAD(P)H dehydrogenase quinone 1; Nrf-2, nuclear factor erythroid 2-related factor 2; ROS, reactive oxygen species; LSE, *Lonicera hypoglauca* and *Scutellaria baicalensis* extract; SOD, superoxide dismutase; SOD1, superoxide dismutase 1; T-AOC, total antioxidant capacity; TNBS, 2,4,6-trinitrobenzene sulfonic acid; TJ proteins, tight junction proteins; TNF-α, tumor necrosis factor alpha-α; UC, ulcerative colitis; ZO-1, zonula occludens-1.
