# Supplementary material for: Supplementation With Chinese Medicinal Plant Extracts From Lonicera hypoglauca and Scutellaria baicalensis Mitigates Colonic Inflammation by Regulating Oxidative Stress and Gut Microbiota in a Colitis Mouse Model
Source: Front Cell Infect Microbiol. 2022 Jan 4;11:798052. doi: 10.3389/fcimb.2021.798052 (PMC8763710; doi:10.3389/fcimb.2021.798052)
Supplement: Supplementary file 1 [file DataSheet_1.docx]

**SUPPLEMENTAL FIGURE LEGENDS**


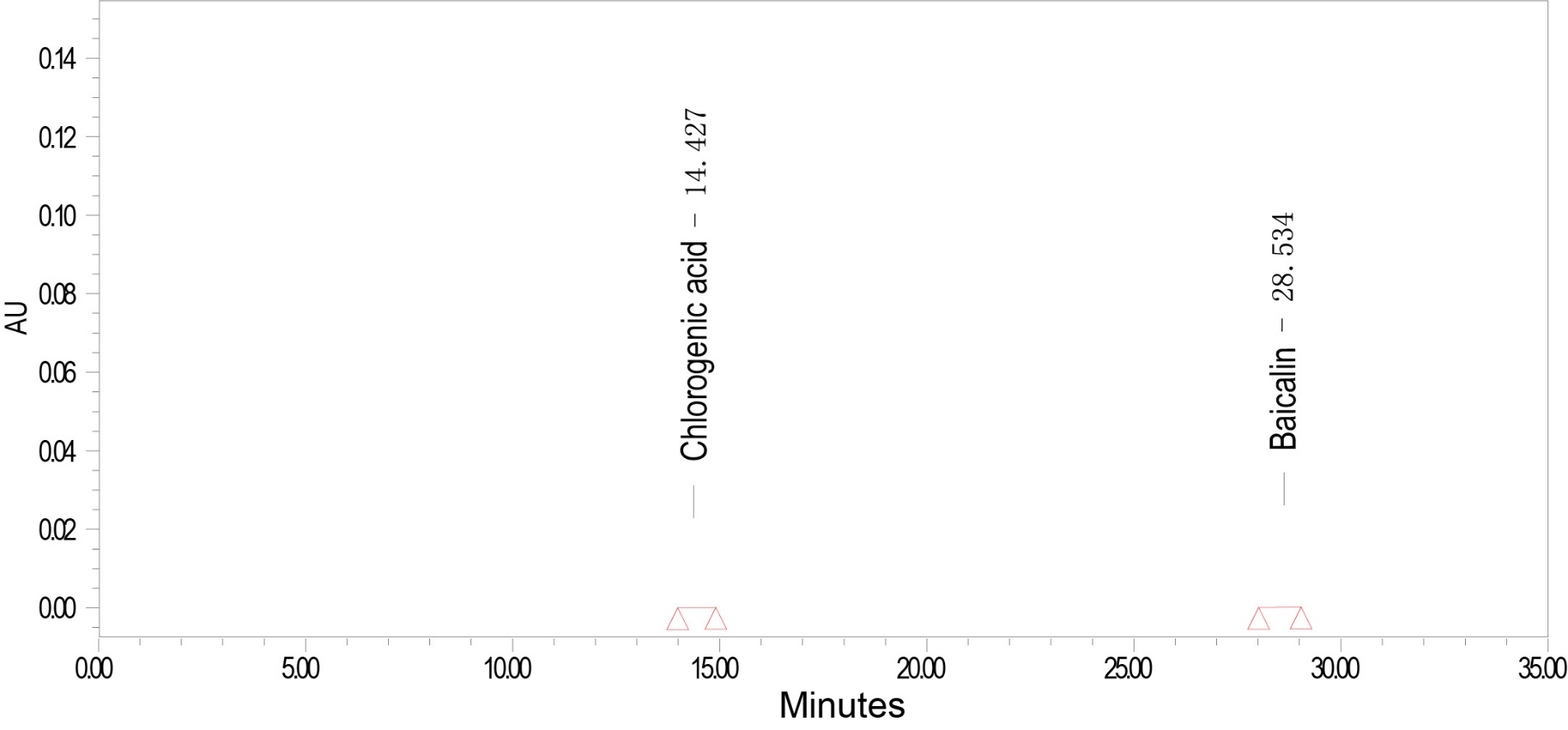
**Figure S1 |** Total ion chromatography (TIC) of LSE.


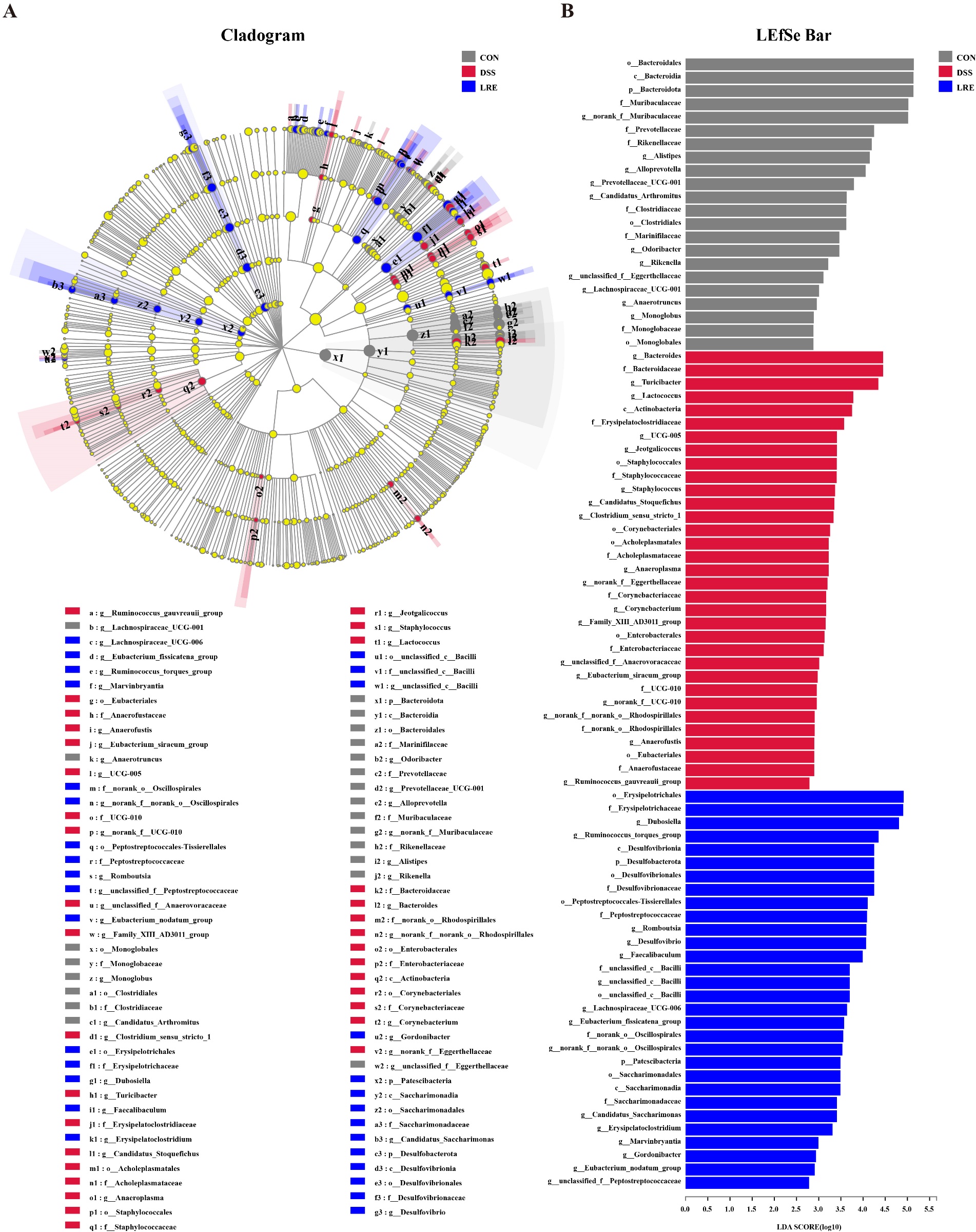
**Figure S2 |** Effect of LRE administration on the colonic microbial composition in DSS-induced colitis mice. (A, B) LEfSe analysis.
